# The p53 Codon 72 PRO/PRO Genotype May Be Associated with Initial Central Visual Field Defects in Caucasians with Primary Open Angle Glaucoma

**DOI:** 10.1371/journal.pone.0045613

**Published:** 2012-09-26

**Authors:** Janey L. Wiggs, Alex W. Hewitt, Bao Jian Fan, Dan Yi Wang, Dayse R. Figueiredo Sena, Colm O’Brien, Anthony Realini, Jamie E. Craig, David P. Dimasi, David A. Mackey, Jonathan L. Haines, Louis R. Pasquale

**Affiliations:** 1 Department of Ophthalmology, Harvard Medical School, Massachusetts Eye and Ear Infirmary, Boston, Massachusetts, United States of America; 2 Centre for Eye Research Australia, University of Melbourne, Royal Victorian Eye and Ear Hospital, Melbourne, Australia; 3 School of Medicine and Medical Science, University College of Dublin, Dublin, Ireland; 4 Department of Ophthalmology, West Virginia University School of Medicine, Morgantown, West Virginia, United States of America; 5 Department of Ophthalmology, Flinders University, Flinders Medical Centre, Adelaide, Australia; 6 Centre for Ophthalmology and Visual Science, University of Western Australia, Lions Eye Institute, Perth, Australia; 7 Center for Human Genetics Research, Vanderbilt University School of Medicine, Nashville, Tennessee, United States of America; Radboud University Nijmegen Medical Centre, The Netherlands

## Abstract

**Background:**

Loss of vision in glaucoma is due to apoptotic retinal ganglion cell loss. While *p53* modulates apoptosis, gene association studies between *p53* variants and glaucoma have been inconsistent. In this study we evaluate the association between a *p53* variant functionally known to influence apoptosis (codon 72 Pro/Arg) and the subset of primary open angle glaucoma (POAG) patients with early loss of central visual field.

**Methods:**

Genotypes for the p53 codon 72 polymorphism (Pro/Arg) were obtained for 264 POAG patients and 400 controls from the U.S. and in replication studies for 308 POAG patients and 178 controls from Australia (GIST). The glaucoma patients were divided into two groups according to location of initial visual field defect (either paracentral or peripheral). All cases and controls were Caucasian with European ancestry.

**Results:**

The p53-PRO/PRO genotype was more frequent in the U.S. POAG patients with early visual field defects in the paracentral regions compared with those in the peripheral regions or control group (p = 2.7×10^−5^). We replicated this finding in the GIST cohort (p  = 7.3×10^−3^, and in the pooled sample (p = 6.6×10^−7^) and in a meta-analysis of both the US and GIST datasets (1.3×10^−6^, OR 2.17 (1.58–2.98 for the PRO allele).

**Conclusions:**

These results suggest that the p53 codon 72 PRO/PRO genotype is potentially associated with early paracentral visual field defects in primary open-angle glaucoma patients.

## Introduction

Adult-onset primary open-angle glaucoma (POAG) is characterized by an irreversible degeneration of the optic nerve that is a common cause of blindness worldwide. POAG is phenotypically and genetically complex and it is likely that multiple genetic and environmental factors play a role in its etiology. Elevated intraocular pressure (IOP) is a major risk factor for optic nerve disease in glaucoma; however, most ocular hypertensive patients do not develop optic nerve degeneration [1] and a number of studies show that POAG patients can develop optic nerve disease despite IOPs in the normal range [2]. Randomized clinical trials, including the Collaborative Normal-tension Glaucoma Study report that lowering IOP does not always result in prevention of progressive visual loss [3]. Collectively these results suggest that some glaucoma patients have a greater susceptibility to optic nerve disease than others. The inherent optic nerve susceptibility exhibited by glaucoma patients may be influenced by a specific set of genetic and/or environmental risk factors, which could be new therapeutic targets for this disease.

Visual field defects in glaucoma patients frequently develop in the periphery and gradually extend to the central region. In a subset of patients the initial functional defect appears in the central visual field as a paracentral scotoma, representing a loss of the maculopapillary nerve fiber layer bundles. Glaucoma patients presenting with early-stage paracentral scotomas are more likely to have systemic risk factors for glaucoma (hypotension, migraine, Raynaud’s phenomenon, and sleep apnea) [4] are more likely to become blind from glaucoma [5] and have lower intraocular pressures than patients with initial defects in the periphery [4], [6]. Together, these observations suggest that formation of paracentral scotomas at early stages of the disease characterizes a subtype of glaucoma, or glaucoma endophenotype, likely to be dependent on a specific set of genetic and environmental risk factors.

Loss of retinal ganglion cells in glaucoma is dependent on the balance of cellular pro-survival and pro-death pathways. Proteins that participate in these pathways are excellent candidates for factors that could influence the susceptibility of retinal ganglion cells to glaucoma-related apoptosis. One of the most important apoptotic regulatory proteins is the tumor suppressor protein p53, which responds to diverse cellular stresses to regulate cell cycle arrest, apoptosis, senescence, and DNA repair [7]. *TP53* (coding for p53) is expressed in retinal ganglion cells under conditions that would stimulate apoptosis [8]–[10] and in an experimental model of glaucoma [11]. Family-based linkage studies have provided evidence for a glaucoma locus on chromosome 17 p that includes *TP53* [12], [13].

*TP53* has a common DNA sequence polymorphism that results in either proline (p53-PRO) or arginine (p53-ARG) at amino-acid position 72 (dbSNP: rs1042522) in the p53 protein. This polymorphism occurs in the p53 proline-rich PXXP domain, which is necessary for the protein to fully induce apoptosis [14]. The proline and arginine variants significantly affect the biological activity of p53, although these effects are highly dependent on the conditions of the study. p53-PRO has increased apoptotic activity in cancer cells in hypoxic conditions [15] and is associated with age-dependent senescence in cultured fibroblasts [16]. p53-ARG increases transcriptional activity of apoptotic related genes in human osteosarcoma cell lines and has higher apoptotic activity in most tumor cells [17]. The apoptotic activity of the codon 72 variants in retinal ganglion cells or other cell types involved in glaucoma is not known.

The p53 codon 72 polymorphism has been studied previously as a risk factor for glaucoma and a consistent association has not been found [18]–[28]. The lack of consistent findings among these studies could be due to variable sample size, ascertainment methodologies, glaucoma case definitions, genotyping methods, and variation in genotypic and allelic frequencies related to population substructure. The p53-PRO allele is the ancestral allele in the African population, while the p53-ARG allele is much more common in European populations [29].

In this study we examine the association between POAG and the p53 codon 72 polymorphism in a Caucasian cohort of European ancestry from the United States and replicate our findings in an independent Caucasian cohort of European ancestry from Australia. The results of our study suggest that the p53-PRO/PRO genotype is potentially associated with a specific glaucoma endophenotype that includes paracentral scotoma formation at an early stage in the disease.

## Materials and Methods

### Participants

The tenets of Helsinki were adhered to and ethics approval was obtained from the Massachusetts Eye and Ear Infirmary (MEEI) institutional review board, and the ethics committees of the Royal Hobart Hospital and the Royal Victorian Eye and Ear Hospital. Written informed consent was obtained from all study participants at both the Massachusetts Eye and Ear Infirmary and the Royal Victorian Eye and Ear Hospital.

264 patients affected with adult onset primary open angle glaucoma (POAG) [64 patients affected with normal tension glaucoma (NTG) and 200 with HT-POAG (‘high-tension’-POAG)], and 400 unaffected individuals were recruited from the Glaucoma Consultation Service and the Comprehensive Ophthalmology Service at the Massachusetts Eye and Ear Infirmary. An additional 308 unrelated people with POAG and 178 controls were recruited through the Glaucoma Inheritance Study in Tasmania (GIST). GIST is derived from a Caucasian population of European ancestry in southeastern Australia, and specific features of this glaucoma cohort have been described previously [23]. All participants had a complete ophthalmological examination, including funduscopic evaluation of the retina and slit-lamp evaluation of the lens. Any patient with retinal or lenticular pathology that could confound the visual field analysis was not included in this study. All of the study participants (all cases and all controls) from both the U.S. and GIST cohorts are Caucasian with reported European ancestry. The features of the cases and controls for both cohorts are presented in Table 1.

**Table 1:** Demographic features of the cases and controls.

Cohort	Group	N	Female (%)	Age at diagnosis*(year)		Maximum IOP(mm Hg)	
				Range	Mean±SD	Range	Mean±SD
MEEI	All glaucoma	264	51.5	32–86	61.3±11.3‡	9–50	24.8±5.9‡
	HT-POAG	200	47.5	32–86	61.4±11.3‡	9–50	26.3±5.5‡
	NTG	64	64.1†	34–83	61.0±11.6‡	13–21	17.9±2.2
	PS	67	59.7	32–83	60.0±11.2‡	9–42	23.8±5.5‡
	NS	147	52.4	34–86	62.0±11.4‡	12–38	24.6±5.6‡
	Controls	400	55.3	39–92	66.0±11.3	9–22	16.1+2.6
GIST	All glaucoma	308	60.7‡	24–89	63.1±12.4	10–74	25.0+9.4
	PS	26	76.9	41–80	63.0±10.5	18–36	24.0+5.2
	NS	152	60.5	24–89	64.2±12.8	10–68	24.2+8.8
	Controls	178	70.2	17–95	65.7±22.3	NA	NA
Pooled	All glaucoma	572	56.5	24–89	62.2±11.9‡		
	PS	93	64.5	32–83	60.8±11.1‡		
	NS	299	56.5	24–89	63.1±12.1‡		
	Controls	578	59.9	17–95	65.9±15.5		

* For controls, this refers to age at enrollment.

† *p*  = 0.02 compared to POAG.

‡ *p*<0.05 compared to controls.

Abbreviations: MEEI, Massachusetts Eye and Ear Infirmary; GIST, Glaucoma Inheritance Study in Tasmania; IOP, Intraocular pressure; HT-POAG, High-tension POAG; POAG, Primary Open Angle Glaucoma; NTG, Normal Tension Glaucoma; PS, Paracentral scotoma; NS, Nasal step/arcuate scotoma; NA, Not available; SD, standard deviation.

Only subjects older than 35 years of age were included in this analysis. HT-POAG was defined as an IOP greater than or equal to 22 mm Hg in both eyes, glaucomatous optic nerve damage in both eyes, and visual field loss in at least one eye. Normal tension glaucoma (NTG) patients had evidence of optic nerve disease and visual field defects with IOP less than 22 mm Hg. Intraocular pressure was measured with a Goldmann tonometer in the clinical setting during typical clinic hours (9 AM to 5 PM). The recorded IOP for each patient was the highest known IOP prior to treatment. Controls had IOP less than 22 mmHg, normal optic nerves and no family history of glaucoma.

### Visual Field Scoring

**Figure 1:**
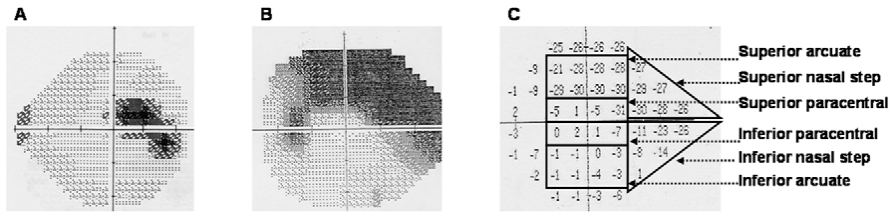
Visual field scoring. The average pattern standard deviation (PSD) in each of 6 regions of the visual field corresponding to the superior paracentral, inferior paracentral, superior nasal arcuate, inferior nasal arcuate, superior nasal step, and inferior nasal step were calculated for each individual. Patients were classified as having paracentral scotomas (PS) if they had a mean PSD in one of the paracentral regions greater than 5 dB and also if the mean PSD was more than 5 dB greater than any other visual field region. Patients were classified as having nasal step/arcuate scotomas (NS) if one of the four possible nasal step or arcuate regions had a mean PSD greater than 5 dB and if one of the four possible regions was 5 dB greater than either of the paracentral regions. The visual field in panel A is an example of a PS, and panel B an example of an NS visual field. The PSD plot in panel C corresponds to the visual field in panel B.

The earliest reliable visual fields demonstrating reproducible defects on at least two independent tests were selected for each affected individual. For this analysis we only used Humphrey automated visual fields (Humphrey Instruments, San Leandro, California, USA) using either the Standard full threshold or Fast-Pac programs. To be included in the study the false positive and negative error rates were less than 20% and the fixation losses were less than 33%. Visual fields demonstrating advanced disease, or generalized depression (mean defect >12 dB) were not used for this analysis. The average pattern deviation in each of 6 regions of the visual field corresponding to the superior paracentral, inferior paracentral, superior nasal arcuate, inferior nasal arcuate, superior nasal step, and inferior nasal step were calculated for each individual (Figure 1). Patients were classified as having an early-stage paracentral scotoma (PS) if they had a focal mean pattern deviation score (PSD) in one of the paracentral regions (either superior or inferior) greater than 5 dB and also if the focal mean pattern deviation score, in at least one of the paracentral regions, was more than 5 dB greater than any other visual field region. Patients were classified as having nasal step/arcuate scotomas (NS) if one of the four possible nasal step or arcuate regions had a mean PSD greater than 5 dB and if one of the four possible regions was 5 dB greater than either of the paracentral regions. Individual eyes were scored independently, and one eye from each patient was used for the analysis. If both eyes qualified for the study, the eye demonstrating the earliest reproducible defect was chosen. 214 visual fields for the MEEI cohort and 178 visual fields for the GIST cohort met these criteria and were used for this analysis.

### Genotyping

DNA was extracted from either peripheral blood samples or mouthwash samples according to previously published protocols [30]. A region of exon 4 of the p53 gene containing the p53 codon 72 polymorphism was amplified and sequenced in all patients using an ABI310 automated sequencer and BIGDYE sequencing chemistry.

### Statistical Analysis

Association analysis was performed using PLINK [31]. Hardy-Weinberg equilibrium was assessed using the chi-squared test. Genotype and allele frequencies of the *TP53* codon 72 variant between patients with glaucoma and control subjects were compared using the Fisher’s exact test. Age-adjusted OR and 95%CI were calculated using logistic regression after adjusting for age at diagnosis for patients with glaucoma and age at enrollment for control subjects. The heterogeneity of ORs between cohorts was evaluated using the Breslow-Day test. Meta-analysis was performed across cohorts assuming a fixed-effect. Multiple comparisons were corrected using the Bonferroni method.

## Results

Because previous studies suggested that p53 could contribute to the development of normal-tension glaucoma [4], [32], we first evaluated the association of the p53 codon 72 polymorphism in the MEEI glaucoma cases with normal tension glaucoma (NTG) defined as IOP less than 22 at time of diagnosis, as well as in the MEEI POAG patients with IOPs at diagnosis equal to or greater than 22 (HT-POAG). The p53-PRO/PRO (proline/proline) genotype and proline allele was associated with NTG overall (p = 0.008 and 0.016 respectively), and nominally with POAG overall (NTG and HT-POAG combined) (p = 0.032 and 0.057 respectively) but not with HT-POAG (p = 0.26) in the MEEI sample (Table 2). Since the p53-PRO/PRO genotype showed more evidence of association with NTG in the MEEI sample and previous studies have suggested that NTG patients are more likely to develop paracentral visual field defects at an early stage of the disease [4], [6], we examined the association between p53-PRO and early paracentral visual field defects in the overall POAG sample (both the NTG and HT-POAG patients).

**Table 2:** Association of p53 codon 72 variant with NTG in the US (MEEI) cohort.

Group	N	Genotype Frequency (%)				Allele Frequency (%)				
		CC	CG	GG	*p* *	C	G	*p* *	OR (95%CI)†	Age-adjusted OR (95%CI)†
All glaucoma	264	26(9.9)	120(45.4)	118(44.7)	0.032	172(32.6)	356(67.4)	0.057	1.27(1.00–1.61)	1.33(1.03–1.73)
HT-POAG	200	16(8.0)	91(45.5)	93(46.5)	0.26	123(30.8)	277(69.3)	0.28	1.16(0.89–1.51)	1.20(0.90–1.59)
NTG	64	10(15.6)	29(45.3)	25(39.1)	0.003	49(38.3)	79(61.7)	0.016	1.63(1.10–2.40)	1.88(1.18–2.98)
Controls	400	19(4.8)	183(45.8)	198(49.5)		221(27.6)	579(72.4)			

* *p* values are calculated using Fisher’s exact test when compared to controls.

† OR and 95% CI are calculated using logistic regression when compared to controls.

Abbreviations: US, United States; MEEI, Massachusetts Eye and Ear Infirmary; POAG, Primary Open Angle Glaucoma; HT-POAG, High-tension glaucoma; NTG, Normal Tension Glaucoma.

We analyzed the earliest available visual fields that demonstrated a reliable significant defect (Figure 1) in the US cohort to assess the association between the p53 codon72 polymorphism and POAG stratified by pattern of visual field loss. 214 MEEI POAG patients had qualifying visual fields and of these 67 had early-stage paracentral defects. The p53-PRO/PRO genotype was more frequent in the paracentral (PS) group (0.22) than in the nasal step/arcuate (NS) group (0.04) or control group (0.05), p = 2.7×10^−5^ (Table 3). The odds of the C allele at p53-PRO was over 2-fold higher in the PS group (p = 4.8×10^−4^, age-adjusted OR 2.20, 95%CI [1.43–3.39]) than in controls.

**Table 3:** Association of p53 codon 72 variants with paracentral scotomas (PS) in the US and GIST cohorts.

			*Genotype Frequency (%)*				*Allele Frequency (%)*						
		N	CC	CG	GG	*p* *	C	G	*p* *	*OR (95%CI)* †	*Age-adjusted OR (95%CI)* †	*p-meta* ‡	*Common OR* *(95%CI)*
US	All glaucoma	264	26(9.9)	120(45.4)	118(44.7)	0.034	172(32.6)	356(67.4)	0.057	1.27(1.00–1.61)	1.33(1.03–1.73)		
	PS	67	15(22.4)	28(41.8)	24(35.8)	2.7×10^−5^	58(43.3)	76(56.7)	4.8×10^−4^	2.00(1.37–2.91)	2.20(1.43–3.39)		
	NS	147	6(4.1)	68(46.3)	73(49.6)	0.99	80(27.2)	214(72.8)	0.94	0.98(0.73–1.32)	0.98(0.70–1.36)		
	Controls	400	19(4.8)	183(45.8)	198(49.5)		221(27.6)	579(72.4)					
GIST	All glaucoma	308	26(8.4)	118(38.3)	164(53.3)	0.25	170(27.6)	446(72.4)	0.11	1.29(0.95–1.75)	1.24(0.91–1.67)		
	PS	26	6(23.1)	11(42.3)	9(34.6)	7.3×10^−3^	23(44.2)	29(55.8)	1.8×10^−3^	2.69(1.48–4.91)	2.32(1.24–4.34)		
	NS	152	8(5.3)	60(39.5)	84(55.2)	0.36	76(25.0)	228(75.0)	0.52	1.13(0.79–1.62)	1.09(0.75–1.57)		
	Controls	178	12(6.7)	57(32.0)	109(61.2)		81(22.8)	275(77.2)					
Pooled‡	All glaucoma	572	52(9.1)	238(41.6)	282(49.3)	0.041	342(29.9)	802(70.1)	0.046	1.21(1.01–1.45)	1.21(1.00–1.46)	0.011	1.28(1.06–1.54)
	PS	93	21(22.6)	39(41.9)	33(35.5)	6.6×10^−7^	81(43.6)	105(56.4)	2.9×10^−6^	2.18(1.59–3.00)	2.23(1.57–3.16)	1.3×10^−6^	2.17(1.58–2.98)
	NS	299	14(4.7)	128(42.8)	157(52.5)	0.90	156(26.1)	442(73.9)	0.99	1.00(0.80–1.25)	0.97(0.76–1.24)	0.74	1.04(0.83–1.31)
	Controls	578	31(5.4)	240(41.5)	307(53.1)		302(26.1)	854(73.9)					

* *p* values are calculated using Fisher’s exact test when compared to controls.

† OR and 95% CI are calculated using logistic regression when compared to controls.

‡ p-heterogeneity >0.05 (Breslow-Day test between cohorts). Meta-analysis assumed a fixed-effect, and common OR and 95%CI are calculated using the Mantel-Haenszel method.

Abbreviations: US, United States; GIST, Glaucoma Inheritance Study in Tasmania; PS, Paracentral Scotoma; NS, Nasal Step/Arcuate Scotoma.

To replicate this finding in a second independent sample we scored Humphrey visual fields for the GIST POAG cohort and evaluated the distribution of p53 alleles in the PS and NS groups. 178 GIST patients had qualifying visual fields and of these 26 had early-stage paracentral defects. In this sample the p53-PRO/PRO genotype was also more frequent in the PS group (0.23) compared with the NS group (0.05) and controls (0.07), (p = 7.3×10^−3^), and the p53-PRO C allele was also more common in the PS group (p = 1.8×10−3, age-adjusted OR 2.32, 95% CI [1.24–4.34]) (Table 3). After testing for heterogeneity we pooled the samples and performed a meta-analysis. We found an overall association of the p53-PRO/PRO genotype with PS in the pooled sample (p = 6.6×10^−7^) and with the PRO allele in the meta-analysis (1.3×10^−6^, OR 2.17, 95% CI [1.58–2.98]). Stratification by gender prior to analysis showed statistically significant association between p53-PRO/PRO and paracentral scotoma formation in both males and females with a somewhat stronger association in males (p = 1.1×10^−4^ in males vs p = 1.8×10^−3^ in females; Table 4). For the p53-PRO/PRO genotype the sensitivity and specificity to detect early-stage paracentral scotoma formation in open angle glaucoma patients is 0.22 and 0.95 respectively with a positive predictive value of 0.6, and a negative predictive value of 0.80.

**Table 4:** Association of p53 codon 72 variants with paracentral scotomas (PS) in the US and GIST cohorts stratified by gender.

				*Genotype Frequency (%)*				*Allele Frequency (%)*						
			N	CC	CG	GG	*p* *	C	G	*p* *	*OR* *(95%CI)* †	*Age-adjusted OR (95%CI)* †	*p-meta* ‡	*Common OR* *(95%CI)*
US	Male	PS	27	7(25.9)	13(48.2)	7(25.9)	0.0010	27(50.0)	27(50.0)	0.0012	2.73(1.52–4.89)	3.15(1.61–6.18)		
		Controls	179	9(5.0)	78(43.6)	92(51.4)		96(26.8)	262(73.2)					
	Female	PS	40	8(20.0)	15(37.5)	17(42.5)	0.0051	31(38.8)	49(61.2)	0.064	1.60(0.98–2.63)	1.68(0.95–3.00)		
		Controls	221	10(4.5)	105(47.5)	106(48.0)		125(28.3)	317(71.7)					
GIST	Male	PS	6	3(50.0)	1(16.7)	2(33.3)	0.035	7(58.3)	5(41.7)	0.036	4.31(1.26–14.74)	2.27(0.62–8.36)		
		Controls	53	5(9.4)	16(30.2)	32(60.4)		26(24.5)	80(75.5)					
	Female	PS	20	3(15.0)	10(50.0)	7(35.0)	0.041	16(40.0)	24(60.0)	0.018	2.36(1.17–4.76)	2.45(1.18–5.11)		
		Controls	125	7(5.6)	41(32.8)	77(61.6)		55(22.0)	195(78.0)					
Pooled‡	Male	PS	33	10(30.3)	14(42.4)	9(27.3)	1.1×10^−4^	34(51.5)	32(48.5)	7.1×10^−5^	2.98(1.76–5.04)	2.90(1.62–5.21)	2.8×10^−5^	2.96(1.75–5.01)
		Controls	232	14(6.0)	94(40.5)	124(53.5)		122(26.3)	342(73.7)					
	Female	PS	60	11(18.3)	25(41.7)	24(40.0)	0.0018	47(39.2)	73(60.8)	0.0041	1.83(1.22–2.74)	1.89(1.21–2.95)	0.0034	1.82(1.21–2.72)
		Controls	346	17(4.9)	146(42.2)	183(52.9)		180(26.0)	512(74.0)					

* *p* values are calculated using Fisher’s exact test when compared to controls.

† OR and 95%CI are calculated using logistic regression when compared to controls.

‡ p-heterogeneity >0.05 (Breslow-Day test between cohorts). Meta-analysis assumed a fixed-effect, and common OR and 95% CI are calculated using the Mantel-Haenszel method.

Abbreviations: US, United States; GIST, Glaucoma Inheritance Study in Tasmania; PS, Paracentral Scotoma.

## Discussion

The results of this study suggest that the p53 codon 72 PRO/PRO genotype is potentially associated with early-stage paracentral visual field defects in patients with open-angle glaucoma. This is the first study to assess association of p53 with glaucoma subsets defined by visual field stratification. Our subgroup analysis could suggest that the previously observed inconsistent association between p53 codon 72 and glaucoma may reflect differences in the composition of the study cohorts with respect to early-onset paracentral cases due to differences in ascertainment methodologies and case definitions. The significant association between the p53 variant and paracentral visual loss among POAG cases vs controls that we discovered in our US cohort was confirmed in the Australian cohort. These results could point to another allele in linkage disequilibrium with the p53 codon 72 variant that is biologically significant; however, considering the known biological activity of the codon 72 polymorphism this possibility seems less likely.

The p53 codon 72 polymorphism influences apoptosis, and the apoptotic potential of the different forms of the protein is dependent on the cell type and cellular environment. p53 activity is determined by the mix of p53 activators and stimulators present in a particular cell type [33]. Stimulators and inhibitors bind specifically to the PXXP motif that contains the codon 72 polymorphic site, and p53-PRO binds both inhibitors and stimulators more efficiently than p53-ARG [30]. Depending on the relative amounts of inhibitors and stimulators the p53-PRO form may have more or less apoptotic activity than p53-ARG. *In vitro* studies using a variety of tumor cells have suggested that p53-ARG has more apoptotic activity than p53-PRO, including a more effective response to oxidative stress and more efficient translocation to the mitochondria [34]. However this relative increase in p53-ARG activity is due to increased activity of the p53 inhibitor iASPP (inhibitory member of the apoptosis-stimulating protein of p53), which reduces the activity of the p53-PRO form but not the p53-ARG form [33]. Interestingly, under conditions of low oxygen tension, the p53-PRO form induces more cell death in cancer cells than p53-ARG [15] and under these conditions the p53 stimulators may be more abundant rendering the p53-PRO form more active. Our results suggest that the p53-PRO has more apoptotic activity in glaucoma, which may reflect variable expression of p53 stimulators and inhibitors in cell types involved in the disease. The p53-PRO apoptotic potential could be enhanced by conditions creating low retinal oxygen tension such as sleep apnea, a condition previously demonstrated to be associated with initial paracentral scotoma formation in glaucoma [4].

Our results could suggest that the ganglion cells in the maculopapillary bundle are more susceptible to p53-mediated apoptosis. The maculopapillary bundle is recognized to be more susceptible to certain metabolic conditions (such as methyl alcohol toxicity) and genetic defects (such OPA1 mutations in dominant optic atrophy) that impair mitochondrial function [35]. The mitochondrial density is higher in the maculopapillary bundle [36] and these cells have higher levels of reactive oxygen species that may lower the apoptotic threshold. The maculopapillary bundle may be more vulnerable to the effects of UV light [37] which could influence mitochondrial function and lower the threshold for apoptosis in carriers of p53-PRO. Early-stage paracentral defects are more common in NTG patients [4] and NTG patients are more likely to have abnormalities of ocular blood flow [38] suggesting that ocular perfusion may also contribute to the increased susceptibility of p53 mediated apoptosis in the paracentral region.

The population frequencies of the PRO/PRO genotype support a role for the p53-PRO allele in glaucoma. p53-ARG is unique to humans, and while the arginine allele is the most common allele in Caucasian populations, the ancestral proline allele is the most common allele in African populations, an ethnic group known to have increased risk of glaucoma [39]. The PRO/PRO genotype is found in 47% of sub-Saharan Africans compared with 8% of European whites. The PRO/PRO genotype is also more common in Japanese (28%) than in European whites, a population with an increased prevalence of normal tension glaucoma [40].

Our results demonstrate a potential association between p53-PRO allele and early-stage paracentral scotoma formation in glaucoma. Several limitations of our study should be considered. First, the number of paracentral cases is low for a formal association study and it is possible that these results could be subject to type II statistical error. Furthermore, while we replicated our findings here, confirmation of these observations in additional datasets will be necessary before a formal association between p53 codon 72 alleles and paracentral scotoma can be established. Second, the low allele frequency and corresponding low sensitivity indicates that testing for the p53-PRO/PRO genotype would not be useful as an overall glaucoma population screening test. The specificity of the association could help identify a subset of glaucoma patients who are at increased risk for early loss of central vision once a formal association is established. Third, these findings are limited to Caucasian patients with European ancestry and may not be generalizable to other populations. Finally, we found that the potential association between p53-PRO/PRO and paracentral scotoma is somewhat stronger in males than in females possibly suggesting that the contribution of p53-PRO is influenced by gender, however further study in other datasets would be required to confirm this observation.

Retinal ganglion cells that degenerate to cause central vision loss may be differentially susceptible to apoptosis and our results suggest that p53 is one protein that could influence this susceptibility. The regulation of glaucomatous ganglion cell apoptosis is clearly complex and it is likely that multiple genetic and environmental factors will contribute to this process. Recent studies have identified a number of genes, in addition to p53, with altered expression in glaucomatous ganglion cells [41], [42]. Examining these genes and their respective pathways will help dissect the underlying genetic causes of early-stage paracentral scotoma formation in glaucoma. Identifying factors that influence the development of paracentral scotomas will be an important step toward preventing visual loss in patients with glaucoma.
